# The adjuvant use of lansoprazole, clonazepam and dimenhydrinate for treating intractable hiccups in a patient with gastritis and reflux esophagitis complicated with myocardial infarction: a case report

**DOI:** 10.1186/1756-0500-6-327

**Published:** 2013-08-16

**Authors:** Georgi Konstantinov Maximov, Deepak Kamnasaran

**Affiliations:** 1 Medical Faculty, Medical University, Sofia, Bulgaria; 2 Department of Pediatrics, Laval University, Québec, QC, Canada

**Keywords:** Intractable hiccups, Myocardial infarction, Lansoprazole, Dimenhydrinate, Clonazepam

## Abstract

**Background:**

Hiccup (Singultus) is a sudden and involuntary contraction of the diaphragm followed by a sharp closure of the epiglottis which results in the production of a specific “hic” sound. Normally, hiccups are treated without intervention. Intractable hiccups occur rarely but are a disturbing symptom underlying other health related disorders.

**Case presentation:**

We report the clinical case of a 67-year-old male patient with myocardial infarction accompanied by intractable hiccups during the course of 8 months, and who was non-responsive to chlorpromazine or metoclopramide, and baclofen; drugs routinely used to treat this condition. This sustained hiccup had severely restricted the patient's ability to intake food and sleep. To explore alternative treatments, we investigated the adjuvant administration of lansoprazole, dimenhydrinate and clonazepam in this patient. We discovered that this drug combination was capable of successfully terminating his intractable hiccups, with no further evidence of recurrence. No similar treatment is previously reported for intractable hiccups. We further suggest a hypothesis concerning a potential mechanism on the anti-hiccup effect of dimenhydrinate.

**Conclusion:**

We identified that the adjuvant use of lansoprazole, clonazepam and dimenhydrinate was capable of attenuating the symptoms of our patient with intractable hiccups.

## Background

Hiccups, also known as ”singultus” or synchronous diaphragmatic flutter means “to gasp” or “sigh” [1,2]. These are involuntary multiple spastic contractions of the diaphragm and intercostal muscles, which are rapidly accompanied by uncontrollable inhalation and a sudden closure of the respiratory tract by the epiglottis, resulting in the classic “hic” sound. Episodes of hiccups involve the unilateral contraction of the left hemi-diaphragm in approximately 80% of cases and with a frequency ranging from 4 – 60 [2]. This phenomenon has little variability among individuals [2], and sometimes both sides of the diaphragm and intercostal muscles are involved [3]. Hiccups are usually benign and self – limited, usually ceasing within minutes and do not require medical treatment [4]. However, hiccups lasting more than 48 hours are categorized as “persistent”, while those lasting more than one month are referred to as ”intractable” [3,5]. Hiccups may persist for months or even years. In fact, severe hiccups are responsible for approximately 4000 annual hospitalizations in the United States [2].

Hiccups may occur at any age including healthy neonates who, in breast feeding, can simultaneously gulp a considerable amount of air into the stomach, which then presses against the diaphragm to produce hiccups. In our lifetime, each of us has experienced hiccups at some time or another. According to the phylogenetic hypothesis, hiccups are evolutionarily antecedent to modern lung respiration; and may potentially explain frequent hiccups among premature infants with pulmonary hypoplasia. Generally, hiccups are harmless and the duration is short. Sometimes these occur for no specific reason, while in other cases, are triggered by a stomach filled with excess food, carbonated beverages or aerophagia, leading to exerting pressure on the diaphragm. Alternatively, the rapid swallowing of food, large volumes of cold beverages with hot meals, very hot or very spicy food, or even excessive amounts of alcohol can induce hiccups. Hiccups can also be triggered by continuous loud laughter, extended inhalation of tobacco or cannabis smoke or even stress among healthy individuals. However, persistent or intractable hiccupps may be accompanied by various serious medical conditions such as myocardial infarction [4,6-11] (Table 1), and therefore warrant pharmacological interventions. In this article, we report the treatment of a patient with intractable hiccups, using lansoprazole, dimenhydrinate and clonazepam, which was successful in attenuating the symptoms with no further evidence of recurrence.

**Table 1:** Medical conditions accompanying persistent or intractable hiccups

	**Medical disorders**
Cardiovascular disorders	myocardial ischemia; angina pectoris; pericarditis [2,3]; myocardial infarction (MI) [2,3,6]; MI as single presenting symptom [4]; internal jugular vein cannulation (as a complication) [7]
Esophago-gastrointestinal disorders	esophageal and gastric distention; erosive esophagitis; herpetic esophagitis; gastro-esophageal reflux; gastric and duodenal ulcers; gastric, pancreatic and colon cancers; hepatoma and liver metastasis; pancreatitis; cholecystitis; ascites; gastric outlet obstruction; small bowel obstruction; subdiaphragmatic abscess; gastrointestinal investigations [2,3]
Central nervous system disorders	traumatic brain injury; brain abscess; meningitis; brain stem lesion; medulla oblongata lesion; lateral medullary infarction [2,3]; brain stem tumor (as primary manifestation) [8]; multiple sclerosis (as single symptom) [9]
Pulmonary and thoracic disorders	pneumonia; pleuritis; asthma; lung tumors; thoracic herpes zoster; acute negative intrathoracic pressure; mediastinal local nerve compression or infection; bronchoscopy [3,4]
Renal disorders	renal failure; uremia; renal cancer; hypernephroma [3,4]
Electrolyte and metabolic disorders	hyponatremia; hypokaliemia; hypocalcaemia; azotemia; uncontrolled diabetes mellitus [3,4]
Others	local and general anesthesia; alcohol intake; psychogenic origin disorders [3,4]

## Case presentation

The patient was male, 67 years of age, who complained of having intractable hiccups for a period of 8 months which had occurred on the fourth day after reanimation related to acute myocardial infarction. Since then, he had persistent hiccups which impaired his ability to eat and sleep. During this period, he occasionally vomited while the sleep deprivation contributed to having acute weight loss. The patient was diagnosed with gastritis, reflux esophagitis, mitral insufficiency and prostate adenoma. His neurological status showed no abnormalities. In addition, none of his family members had similar symptoms. Treatment with routine medications including those typically prescribed in intractable hiccup cases was as follows: chlorpromazine, metoclopramide and baclofen, and all unfortunately were ineffective in this patient. Therefore, we investigated the adjuvant dose of lansoprazole (15 mg daily), dimenhydrinate (25 mg, trice daily) and clonazepam (0.5 mg, twice daily) on the patient. With this treatment regimen, his intractable hiccups were completely attenuated on the third day with no adverse side-effects documented. No further administration of this drug regimen was necessary, and there was no evidence of symptomatic recurrence.

## Conclusion

Drug treatment is required when hiccups persist longer than normal. However, it must be recognized that some drugs could further aggravate the hiccups [2]. These include, cisplatin, Cyclophosphamid®, carboplatin, docetaxel, paclitaxel, etoposide, Gemcitabine-HCL®, irinotecan, vindesine and Vinorelbin® [2], corticosteroids especially dexamethasone [12], benzodiazepines especially Apo-Diazepam® [2], anabolic steroids [2], opioids particularly morphine [13], heroin methadone, antibiotics especially ceftriaxone [14] and azithromycin [15]. The principal treatment for chronic hiccups is currently with chlorpromazine [16] and metoclopramide. In the United States, chlorpromazine is the only Food and Drug Administration approved drug to treat this symptom [3]. However, other drugs may include the gamma aminobutyric acid agonist baclofen [17] but unfortunately this is intolerable among many elderly patients secondary to adverse side effects [2]. Alternatively, another gamma aminobutyric acid agonist, namely Gabapentin-GR®, a recent anti-epileptic drug, shows improved efficacy and with less side effects when treating intractable hiccups [18,19]. In contrast, the classical anti-epileptic drugs Apo-Carbamazepine®, valproic acid and phenytoin are rarely used because of their adverse drug interactions. Moreover, the dopamine antagonist Apo-Haloperidol®, and the tricyclic anti-depressant amitriptyline, may attenuate hiccups but are associated with significant adverse side effects [2]. Other studies suggest the use of calcium channel blockers nifedipine [20], nimodipine [21] and lidocaine [22], non-opioid analgesic nefopam [23], Amantidine® [24], olanzapine [1], carvedilol [25], Methylphenidate-HCL® [26], all of which may treat chronic hiccups, but warrant further investigation. Interestingly, dexamethasone and midazolam, although recognized to trigger the onset of hiccups, were successfully utilized only in a few cases [2,27,28]. Marijuana, although recognized as an opioid, was previously described to potentially treat intractable hiccups [3,29], but is subject to current controversy. Hence, it is imperative to continue further investigations for the treatment of intractable hiccups.

In our study, the rationale to use lansoprazole was to suppress the patient's symptoms of gastritis and reflux esophagitis. Principally, lansoprazole promotes the healing of gastric and duodenal ulcers and treats gastrointestinal reflux. Lansoprazole, a potent suppressor of gastric acid secretion, is an inhibitor of the gastric H^+^, K^+^ - ATPase (proton pump), which exchanges hydrogen and potassium ions across the parietal cell membrane [30]. The crucial structures for the central nervous system stimulation of gastric acid secretion are the dorsal motor nucleus of the vagal nerve, the hypothalamus and the solitary tract nucleus [30], that predominantly modulate the activity of the enteric nervous system via acetylcholine release from the vagal efferent fibers. Lansoprazole diminishes the daily production of acid (basal and stimulated) by 80% to 95%. In fact, maximal suppression of acid secretion requires several doses of the proton pump inhibitor. For example, it may take 2 to 5 days of therapy with one daily dosage to achieve 70% inhibition of proton pump activity that is seen at a steady state [30]. Furthermore, lansoprazole, like other proton pump inhibitors, is endowed with causing only very few adverse effects.

Patients with intractable hiccups have impaired quality of life leading to episodes of anxiety, depression, sleep deprivation, vomiting, malnutrition, dehydration, weight loss and speech problems [2]; hence requiring the need for anti-anxiety therapy. Currently, benzopdiazepines (especially clonazepam) and selective serotonine re-uptake inhibitors, are most commonly used for treating anxiety disorders. Benzodiazepines are speculated by some gastroenterologists to ameliorate a variety of “anxiety related” gastrointestinal disorders. However, there is a paucity of evidence for direct action. Clonazepam, like other benzodiazepines, exert most of its effects by interacting with the inhibitory neurotransmitter receptors which are directly activated by gamma aminobutyric acid. As the dose of a benzodiazepine is increased, sedation progresses to hypnosis. The half-life of clonazepam in plasma is about 1 day. In our patient, clonazepam was administered in small doses due to its potent myo-relaxant, tranquilizing, anxiolytic properties, and notably, sleep-inducing effects. This ultimately resulted in a minor sedation of the patient, while being adjuvantly treated with dimenhydrinate.

Dimenhydrinate represents a combination of diphenhydramine and 8-chloro theophylline in equal molecular proportions. We administered dimenhydrinate to our patient, due to its property of being a first generation histamine H1 receptors antagonist. H1 receptors are distributed in smooth muscles, endothelial cells and the central nervous system. H1 antagonists, such as dimenhydrinate, inhibit the effect of histamine on these structures. Likewise, it inhibits responses to acetylcholine that is mediated by muscarinic receptors. H1 antagonists do not suppress gastric secretion but are most useful when having sedative properties to treat acute types of allergy and motion sickness. Dimenhydrinate has dual functions of stimulating and depressing the central nervous system. Central excitation is a striking feature of overdose, while central depression on the other hand usually results in diminished alertness and somnolence. Following oral administration, the peak plasma concentrations are achieved within 2 to 3 hours and the effect usually lasting 4 to 6 hours. In our case, the dose of 25 mg, was in compliance with the age of the patient. Our hypothesis, to explain the anti-hiccup effect of dimenhydrinate is based on its property of being a histamine H1 receptors antagonist. Myocardial infarction results in myocardial tissue injury where basic polypeptides like histamine are released. This can cause spasmogenic by the binding of histamine to H1 receptors of the enteric smooth muscles, thereby irritating the afferent branches of the hiccup-reflex arch and subsequently the onset of hiccups. Hence, treatments with dimenhydrinate attenuate this process and can relieve the symptoms of chronic hiccups.

In conclusion, we identified that the adjuvant use of lanzoprazole, clonazepam and dimenhydrinate was capable of terminating the symptoms of intractable hiccups in our patient; findings which will require further investigations among other similar cases, in an effort to discover more effective or improved treatment regimens for intractable hiccups.

## Consent

Written informed consent was obtained from the patient for publication of this Case Report and any accompanying images. A copy of the written consent is available for review by the Editor-in-Chief of this journal.

## Competing interests

The authors declare no conflict of interest.

## Authors’ contributions

GKM: co-wrote manuscript, conceptualized presentation of study design/data analyses, clinically managed patient. DK: wrote manuscript, conceptualized presentation of study design/data analyses. Both authors read and approved the final manuscript.
